# Light-Addressable Potentiometric Sensors in Microfluidics

**DOI:** 10.3389/fbioe.2022.833481

**Published:** 2022-02-21

**Authors:** Xueliang Li, Shibin Liu, Jie Tan, Chunsheng Wu

**Affiliations:** ^1^ School of Mechanical and Electrical Engineering, Zhoukou Normal University, Zhoukou, China; ^2^ College of Electronics and Information, Northwestern Polytechnical University, Xi’an, China; ^3^ Institute of Medical Engineering, Department of Biophysics, School of Basic Medical Sciences, Health Science Center, Xi’an Jiaotong University, Xi’an, China

**Keywords:** light-addressable potentiometric sensor, micropump, microfluidics, chemical sensors, biosensors

## Abstract

The light-addressable potentiometric sensor (LAPS) is an electrochemical sensor based on the field-effect principle of semiconductors. It is able to sense the change of Nernst potential on the sensor surface, and the measuring area can be controlled by the illumination of a movable light. Due to the unique light-addressable ability of the LAPS, the chemical imaging system constructed with the LAPS can realize the two-dimensional image distribution detection of chemical/biomass. In this review, the advantages of the LAPS as a sensing unit of the microelectrochemical analysis system are summarized. Then, the most recent advances in the development of the LAPS analysis system are explained and discussed. In particular, this review focused on the research of ion diffusion, enzymatic reaction, microbial metabolism, and droplet microfluidics using the LAPS analysis system. Finally, the development trends and prospects of the LAPS analysis system are illustrated.

## Introduction

The microfluidic chip is an emerging and powerful analysis technology, which has been widely applied in the development of microanalysis systems. In other words, based on the micro-electro-mechanical system (MEMS) technology, a variety of microstructures such as microchannels, micromixers, microreaction chambers, and micropumps are fabricated on glass, silicon wafers, organic polymers, and other materials. A series of discontinuous processes in biochemical analysis, including sample collection, processing, reaction, and detection, are integrated on a chip to achieve miniaturization and integration of the analysis equipment, which realizes lab-on-a-chip. Therefore, it has the advantages of fast analysis speed, less pollution, parallel processing, etc., and has gradually attracted more and more attention and has been applied in many fields such as clinical examination and environmental monitoring (Whitesides, 2006).

The detector is one of the core components of a microfluidic chip. Since the advent of microfluidic chips, the research on detectors suitable for integration with microfluidic chips has been a hot spot. Among many microfluidic detection technologies such as electrochemical, titration, fluorophotometry, and colorimetry, electrochemical detection has shown many advantages such as a simple structure, small volume, no pollution to samples, and compatible with the micromachining technology, which have incomparable advantages in integration with chips (Yoshinobu et al., 2015), especially field-effect sensors such as the Light-addressable potentiometric sensor (LAPS).

The LAPS is a kind of potentiometric semiconductor chemical/biological sensor, which is developed based on the light-electric effect and field-effect principle in semiconductors. Since the LAPS appeared in the late 1980s it has become one of the research hot spots in the chemical/biological sensor because of its small size, high detection accuracy, flexible use, and strong addressable detection capability. In 1988, Hafeman et al. analyzed the working principle of the LAPS for the first time in *Science*, which pointed out that the LAPS can sensitively detect the weak change of the solid–liquid interface potential on the surface of the LAPS (Hafeman et al., 1988). Therefore, various biochemical reactions such as the enzymatic reaction, redox reaction, and antigen–antibody specific reaction could potentially be detected by the LAPS. At present, the LAPS has broad application prospects in the fields of pH detection (Yang et al., 2020), ion detection (Wen et al., 2016; Zhang et al., 2018), molecular detection (Li et al., 2020; Zhang et al., 2020), enzymatic reaction (Liang et al., 2017), cell metabolism (Dantism et al., 2015; Dantism et al., 2017; Wu et al., 2017; Du et al., 2018; Dantism et al., 2019), immunoassay, and DNA detection (Wu et al., 2015; Jia et al., 2019; Tian et al., 2019).

Comparing to other electrochemical sensors, the LAPS is not only easy to integrate and miniaturize but also can realize label-free and quantitative detection, thus avoiding the side effects of fluorescent reagents on the analytical solutions (Guo et al., 2014). The surface of the LAPS is flat and uniform, and its surface material has the same properties with preparing microchannels. Therefore, it is possible to construct microfluidics with arbitrary shapes on the surface of the LAPS (Wagner et al., 2011). The LAPS is light-addressable; this unique quality makes it possible to detect the chemical/biological information of analytical samples at any position of the microchannels. Moreover, the materials used to prepare the microchannel paths are generally transparent, so the microfluidics constructed by the LAPS as a detection sensor can also be utilized to study some chemical/biological reactions with light characteristics under a light microscope (Yoshinobu et al., 2017). As a result, the LAPS is very suitable as a microfluidics detection sensor.

**FIGURE 1:**
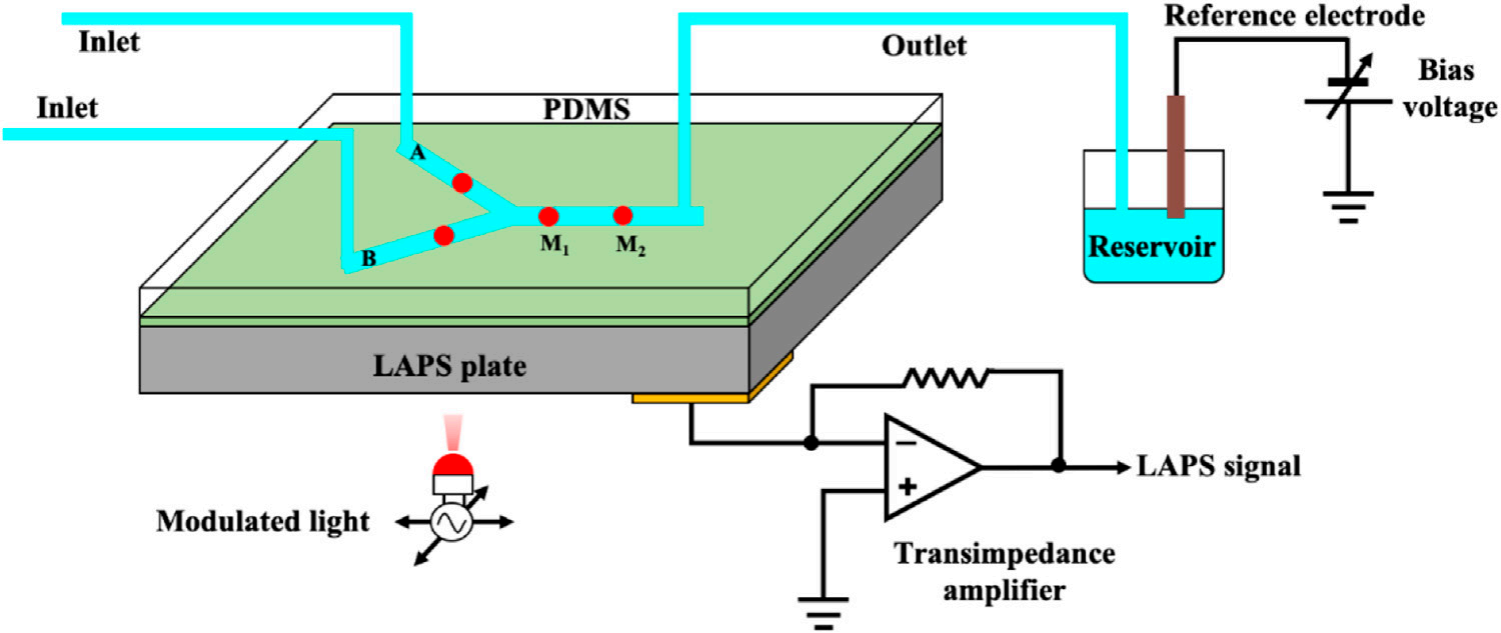
Schematic diagram of the microchannel analysis system based on the LAPS (Wagner et al., 2011).

A schematic diagram of the microchannel analysis system based on the LAPS is shown in Figure 1, which shows that the decisive advantages of the LAPS-based microchannel analysis system include the following: 1) the LAPS can customize the position of sensors with its unique light-addressable function, which enhances the flexibility of the microfluidic chip design and 2) it can track and detect the time-varying information of the concentration of the analytical solution down to nanoliter at any point (for example, the red points in Figure 1) on the microchannel path and 3) the flowrate of the liquid could be down to dozens of nanoliters per second. With these unique advantages, the LAPS has gradually become a promising research topic in the field of microfluidics.

This review mainly summarizes the most recent research progress of the LAPS analysis system. In the second part, the principle of the LAPS and the chemical imaging principle of the LAPS are briefly introduced. The third part summarizes and discusses several applications of the LAPS analysis system. Finally, the future development trends and prospects of an LAPS analysis system are illustrated.

## Principle

### Principle of the LAPS

**FIGURE 2:**
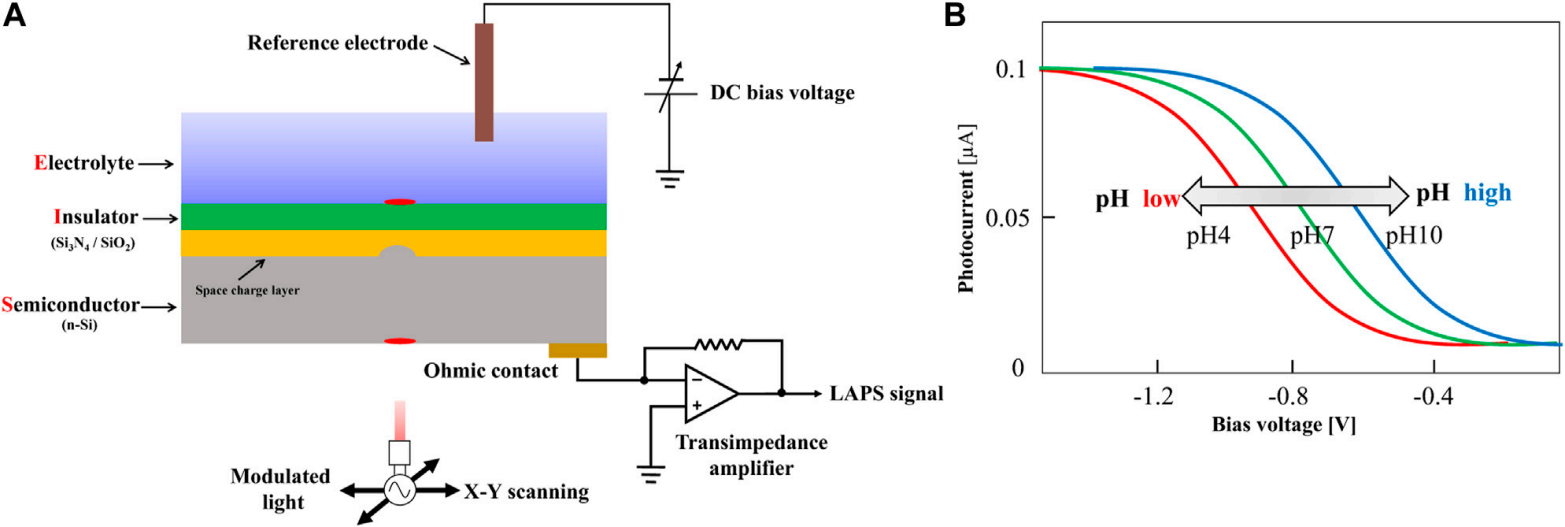
**(A)** Schematic representation of a typical LAPS set-up; **(B)** I-V characteristic curve of the LAPS(Yoshinobu et al., 2015).

The basic structure of the LAPS includes four layers from top to bottom: the sensing layers, insulating layer, semiconductor substrate, and working electrode. Figure 2A shows the structure and working principle diagram of the LAPS, which is featured with an electrolyte–insulator–semiconductor (EIS) stack. In the measurement, taking n-type silicon as an example, a bias voltage is applied to the LAPS chip through a reference electrode and working electrode. By conducting the electrolyte solution (analytical solution), the bias voltage acts on the surface of the LAPS, pushing the majority carriers at the interface between the insulating layer and the silicon substrate to the silicon substrate. Therefore, a space charge region consisting of immobile fixed ions, namely, a depletion layer, is formed at the interface between the insulating layer and the silicon substrate. When the LAPS is illuminated from the back by an AC-modulated light source with a specific wavelength, a large number of photogenerated carriers are excited in the silicon substrate. Then, most of the photogenerated carriers diffuse to the depletion layer generating at the interface at the semiconductor and insulator continuously. Furthermore, in the process of diffusion, some photogenerated minority carriers recombine with majority carriers. Meanwhile, some unbound photogenerated carriers diffuse into the depletion layer, and they are immediately separated by the internal electric field of the depletion layer. As the photogenerated minority carriers accumulate at the interface between the insulating layer and silicon substrate, the photogenerated potential has been formed. Since the intensity of the light source is AC-modulated, the number of photogenerated minority carriers accumulated at the interface between the insulating layer and the silicon substrate also changes dynamically. The variation of photogenerated potential at the interface is synchronized with the modulation signal of the light source, forming only an alternating current (none DC current) in an external measuring circuit for the insulator. It can be detected by using a galvanometer. The number of photogenerated carriers that can diffuse into the depletion layer is very small. So the amplitude of the output weak current signal ranges from tens of nanoamps to tens of microamps (Yoshinobu et al., 2015).

In order to detect the ideal target species, it is necessary to modify the LAPS surface with a sensitive film. The sensitive film is able to generate responsive changes when the target species appears on the sensor surface. For example, the silicon nitride film, which is sensitive to hydrogen ions, is commonly used to modify the LAPS surface to detect hydrogen ions. Basically, by chemical or physical methods, a sensitive film which has potential responses to the species that can be measured in the electrolyte solution will be deposited on the surface of the LAPS insulating layer. In this case, on the surface of the sensitive film, a solid–liquid interface would be formed; it is potentially proportional to the concentration of the measured species in the solution. This potential is superimposed on the external bias voltage, which changes the thickness of the space charge region between the LAPS insulating layer and the silicon substrate. This allows the change of photogenerated potential under the same light source conditions and consequently causes the change of the current in the external measuring circuit. As a result, by recording the changes of the current amplitude, the changes in the concentrations of the measured species in the solution can be measured. Generally, the bias voltage varies linearly within a certain range and measures the output photocurrent of the LAPS at the same time. The plotted photocurrent-bias voltage curve is the *I-V* characteristic of the LAPS, as shown in Figure 2B.

### Chemical Imaging Principle of the LAPS

The LAPS can customize the detection area by changing the illumination position of the light source so that the pH value of any detection point can be measured by the light scanning technology. Based on the light addressable ability of the LAPS, Nakao et al. proposed a chemical image sensor based on the LAPS and obtained the two-dimensional pH chemical image of the solution (Nakao et al., 1994). In addition, it was used to observe the distribution of hydrogen ions in the external microenvironment of living cells. According to the basic principle of the LAPS, the pH value of the solution can be obtained by the offset of the *I-V* characteristic curve. With the decrease in the pH value, the output photocurrent of the LAPS decreases as well. On the other hand, with the increase in the pH value, the output photocurrent of the LAPS also changes accordingly. When the bias voltage is fixed, the detection point of the substrate of the LAPS is illuminated by a light source with a certain illumination area. Therefore, the pH value of the illumination position can be obtained. Then, the pH value is converted to a gray value corresponding to the image. According to this method, the remaining positions of the substrate of the LAPS are irradiated in a certain order, and finally, the chemical distribution image of the solution pH value can be obtained (Liang et al., 2019a).

## Research Progress of the LAPS in a Microelectrochemical Analysis System

Since the LAPS has the decisive advantages of light addressability, flat surface, and simple structure, it has been widely applied in the development of the microanalysis system *via* the integration with a microfluidic chip. More and more attention has been paid to this field, and some exciting progress has been made. This review will focus on the most important and representative examples of LAPS application in microanalysis systems. Therefore, this section summarizes and discusses the applications of LAPS-based microanalysis systems, which include ion diffusion, enzymatic reaction, microbial metabolic activity, and droplet microfluidics. Furthermore, characteristics of the LAPS for a microelectrochemical analysis system with category, system characteristics, and purpose can be seen in Table 1.

**TABLE 1:** Characteristics of the LAPS for a microelectrochemical analysis system with category, system characteristics, purpose, and performance (Selected examples).

Category	System characteristics		Purpose	Performance	Reference
Ion diffusion	LAPS analysis system	Observing ion diffusion between the cathode and anode		the diffusion coefficients for Na+ ions as $1.3\times 10^{-9}m^{2}/s$	Yoshinobu et al. (2000)
	LAPS analysis system with a grating PDMS microchannel	Observing the chemical images of some special ions or macromolecules		multi-ion could be sensing and imaging in a microchannel	Yoshinobu et al. (2005)
	LAPS analysis system with Y−shaped microchannels	Investigating the mechanisms of microfluidics		the diffusion coefficients for Na+ ions as $1.27\times 10^{-9}m^{2}/s$	Miyamoto et al. (2013)
	LAPS analysis system with Y−shaped microchannels	Analyzing the feasibility about constructing a microchannel analysis system on the LAPS surface		the system can address and read out a measurement spot in about 160 ms	Wagner et al. (2011)
	LAPS analysis system with a PDMS microchannel	Observing the ion concentration distribution in the microchannels		64 points realized recording of movies at a frame rate of up to 100 fps	Miyamoto et al. (2014)
Enzymatic reaction and microbial metabolism	LAPS analysis system with a trap structure microchannels	Chemical imaging about enzymatic reaction products		urea concentration in the range of $0.3\times 10^{-3}\;∼10^{-1}mol/L$	Miyamoto et al. (2011)
	LAPS analysis system with the PDMS microchannel	Calculating the number of cells		cell counting and analyzing	Liu et al. (2010)
	LAPS analysis system with a grating PDMS microchannel	Studying cellular metabolic mechanisms and drug reactions		the pH resolution was 0.904 mpH versus 5.434 mpH	Hu et al. (2013)
	LAPS analysis system with bubble-capturing microchannels	Detecting the metabolic rate of liver tumor cells		the pH sensitivity of LAPS is 335.5 nA/pH	Liang et al. (2019b)
	LAPS analysis system with microchannel support cell living	Detecting the signal transduction mechanism of taste cells		around 1/3 bioengineered cells are expressed with bitter receptors	Du et al. (2019)
Microanalysis about digital droplets	LAPS analysis system with valve controlled microchannels	Microdroplet measurement		the minimum volume of the droplet was 400 nl	Miyamoto et al. (2016)
	LAPS analysis system with a electroosmotic micropump microchannel	Microdroplet measurement		the volume of the droplet as low as 1 nl	(Li et al., 2016), (Li et al., 2017)

### Ion Diffusion

The most important characteristic of the LAPS is its ion-sensing capability. By combining the light addressability of the LAPS, an image showing the diffusion of ions on the surface of the LAPS can be obtained. For instance, Yoshinobu et al. reported a miniature LAPS analysis system for studying ion diffusion (Yoshinobu et al., 2000). Through the electrolysis of low-concentration sodium chloride solution, the ion diffusion between the cathode and anode was observed using this microanalysis system. This LAPS analysis system was also used to collect the acid–base concentration distribution images around the electrode, fit the diffusion equation, and calculate the diffusion coefficient and the molecular weight.

Furthermore, the integration of a LAPS chip with a microfluidic chip allows for monitoring the dynamic changes of ions inside the microchannels. The microfluidic chip can precisely control the movement of a small amount of ion solutions inside microchannels, while the LAPS can sensitively detect the concentration of local ions inside microchannels. Therefore, the combination of a microfluidic chip with a LAPS chip provides an ideal platform for the study of ion diffusion. Yoshinobu et al. constructed a grating PDMS microchannel on the surface of the LAPS using soft lithography technology and built a LAPS analysis system by driving the analysis sample with a microinjector. It can observe the chemical images of some special ions or macromolecules (Yoshinobu et al., 2005). The flow of the pH buffer solution in the microchannels can be observed by the microelectrochemical analysis system.

With further development and modification on microchannels, the LAPS-based microanalysis system can also be used to investigate the mechanisms of microfluidics. Miyamoto et al. constructed a LAPS analysis system with a Y-shaped microchannel structure and observed the laminar flow phenomenon in the microchannels (Miyamoto et al., 2013). By studying the ion diffusion law at the interface of two microchannels, the ion diffusion coefficient was determined. The photocurrent distribution of laminar flow in Y-shaped microchannels under different injection flow rates was studied. Therefore, the LAPS analysis system is expected to become a new microchannel analysis platform to study the laminar flow in microfluidics.

The feasibility of constructing a microchannel analysis system on the LAPS surface is demonstrated. For example, Wagner et al. expounded the advantages of constructing the LAPS as a detection sensor to build a microelectrochemical analysis system (Wagner et al., 2011). By constructing a Y-shaped microchannel unit on the surface of the LAPS, the red area represents the measurement point defined by the LAPS sensor, points A and B represent the initial measurement points of two solutions, respectively, and P (0) represents the measurement point when the two solutions are mixed. P (1) represents the measurement point after two solutions are mixed for a period of time. All the measurement points can be freely selected during operation. By attaching a layer of transparent polymer material to the local area of the LAPS surface, the electrical impedance of the detection loop is increased so as to reduce the detection photocurrent. As can be seen from the legend, the additional part of the image is obviously dimmed. Therefore, it is proved that the scheme of constructing a microchannel analysis system with the LAPS as a detection sensor is feasible.

**FIGURE 3:**
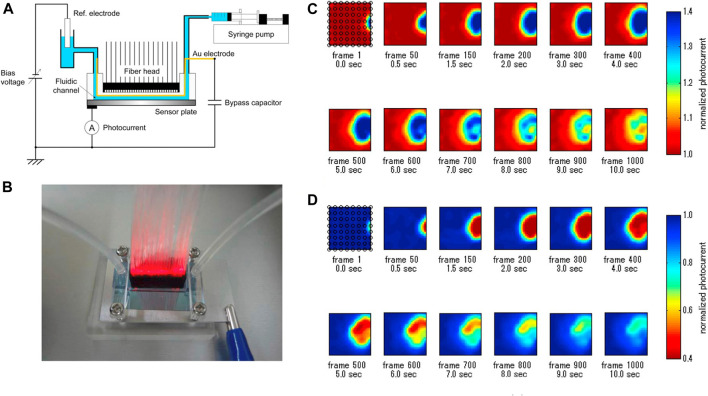
**(A)** Schematic diagram of LAPS-based microfluidics; **(B)** Illumination of the front of the LAPS with a 64-channel light fiber; **(C)** and **(D)** represent chemical images of the injected 10 mM NaOH solution and 10 mM HCl solution, respectively (Miyamoto et al., 2014).

One of the decisive advantages of microfluidic chips originated from the large-scale of its identical units, which makes it possible to achieve high-throughput measurement. For example, Miyamoto et al. reported a LAPS analysis system which can observe the ion concentration distribution in the microchannels (Miyamoto et al., 2014). As shown in Figure 3, by constructing a PDMS microchannel on the surface of the LAPS and using 64 guiding fibers with different frequencies as modulated light sources, the parallel measurement of 64 detection sites was realized. Furthermore, microfluidics was studied by using a syringe pump, and the maximum acquisition speed of the ion concentration image was 100 frames/s. The research results show that the system is expected to be widely used in the field of flow injection analysis, especially in the high-throughput analysis of microdroplets. This is a solid work to demonstrate the fast-imaging speed up to now, which still has the high complexity of instrumentation from multi-frequency modulation and the FFT signal process.

Thanks to the light addressability of the LAPS, it could be used as a microfluidic sensor for imaging of ion diffusion. In the future, how to improve the spatial resolution of the LAPS and reduce the consumption of analytical samples should be considered.

### Enzymatic Reaction

**FIGURE 4:**
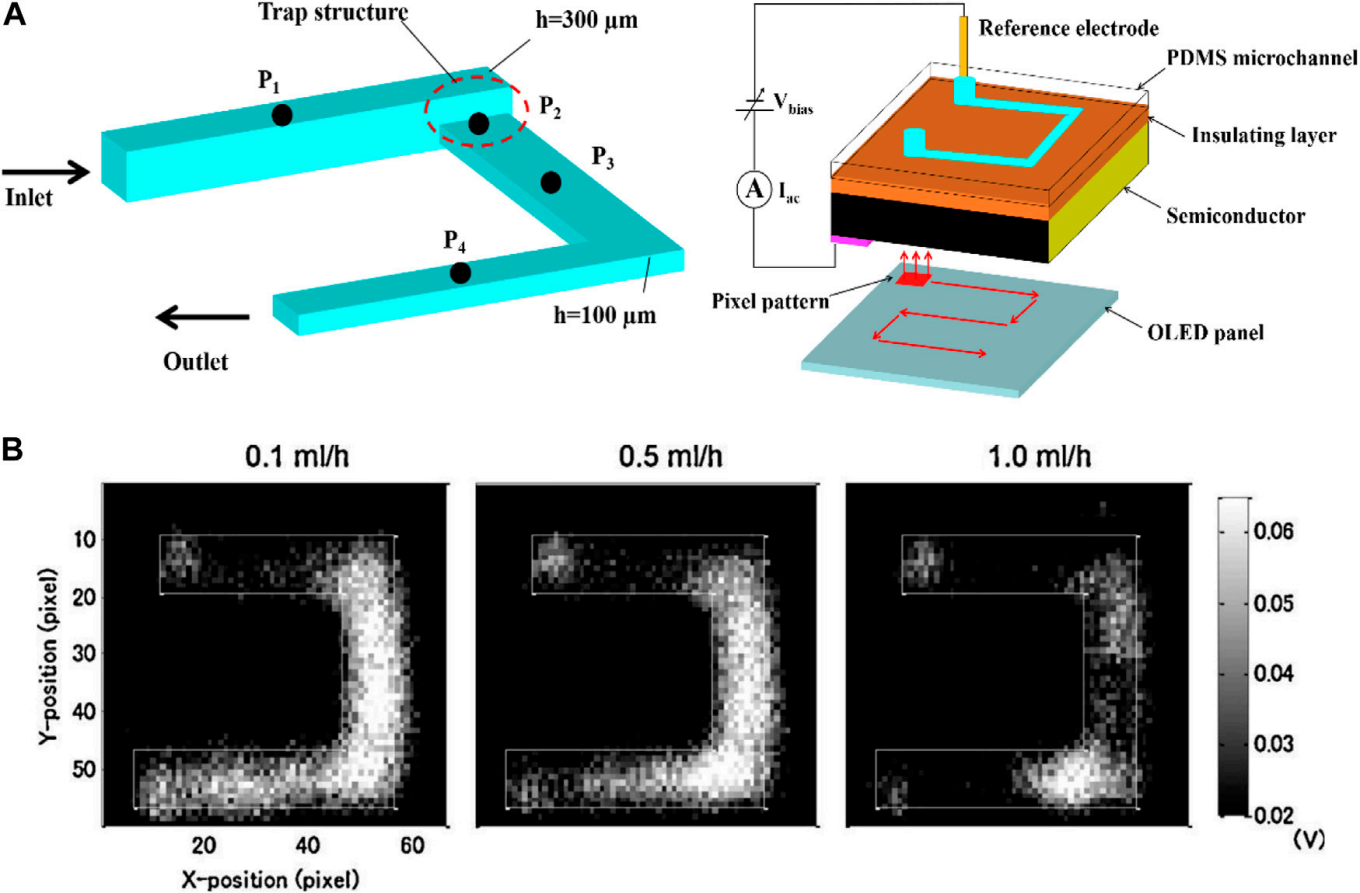
**(A)** Schematic diagram of the U-shaped microchannel LAPS analysis system with a trap structure; **(B)** Distribution of enzymatic reaction images of urea solution in microchannels under different urease injection flow rates, 0.1 ml/h, 0.5 ml/h, and 1 ml/h (Miyamoto et al., 2011).

In addition, the LAPS analysis system also shows broad application prospects in the field of biosensors, especially in the detection of enzymatic reaction products. For instance, Miyamoto et al. constructed a LAPS analysis system for detecting urea reaction products (Miyamoto et al., 2011). Due to the implantation of urease gel microspheres into microchannels, when the urea solution is injected into the microchannels, the pH change of the downstream solution of microchannels can be detected, which can reflect the kinetic process of the enzymatic reaction in the microchannels. Figures 4A,B show the typical pH image distribution in the microchannels with a trap structure and the microchannels, respectively. The potential distribution of the solution is related to the injection flow rate, and it is related to the reaction time of the enzymatic reaction. Therefore, the kinetic process of the enzymatic reaction can be described by the potential distribution of the solution. However, due to the usage of OLED as the light source, the resolution of LAPS maps was not well.

The LAPS analysis system can also realize visual analysis of neurotransmitters such as acetylcholine. Werner et al. constructed a miniature LAPS analysis system by modifying the surface of the LAPS with acetylcholinase and realized the visual analysis of acetylcholine (Werner et al., 2013). Similarly, a CCD-based pH image sensor can also be used for visual analysis of acetylcholine (Takenaga et al., 2012).

### Microbial Metabolism

Metabolic perturbations of living bacterial cells are essential for assessing bacterial and cellular activities, which can usually be measured by reflecting the rate of extracellular acidification. For instance, the activation of receptors can modulate the rate of extracellular acidification by causing metabolic changes that affect the demand for ATP. As a result, bacterial and cellular activities can be evaluated indirectly by measuring extracellular acidification. The LAPS is one of the most commonly used measurement techniques of bacterial and cell metabolism *in vivo*. A traditional LAPS modified with a sensing layer of Si_3_N_4_ has a high sensitivity close to the Nernst limit. Therefore, the LAPS is an ideal device for the measurement of microbial metabolism, especially when it is integrated with microfluidic chips. For example, Liu Qingjun et al. reported a micro LAPS analysis system which can calculate the number of cells (Liu et al., 2010). The PDMS microchannel was prepared by micromachining technology, and the PDMS microchannel was bonded to the surface of the LAPS to form a cross-shaped microchannel on the silicon substrate. The cell culture medium was driven by using a peristaltic pump, and the rat blood cells driven by gravity were counted and analyzed by measuring the LAPS photogenerated current. In this study, microfluidics technology is introduced into the research of the LAPS cell sensor, which promotes the miniaturization and multifunction of a cell-counting analyzer.

**FIGURE 5:**
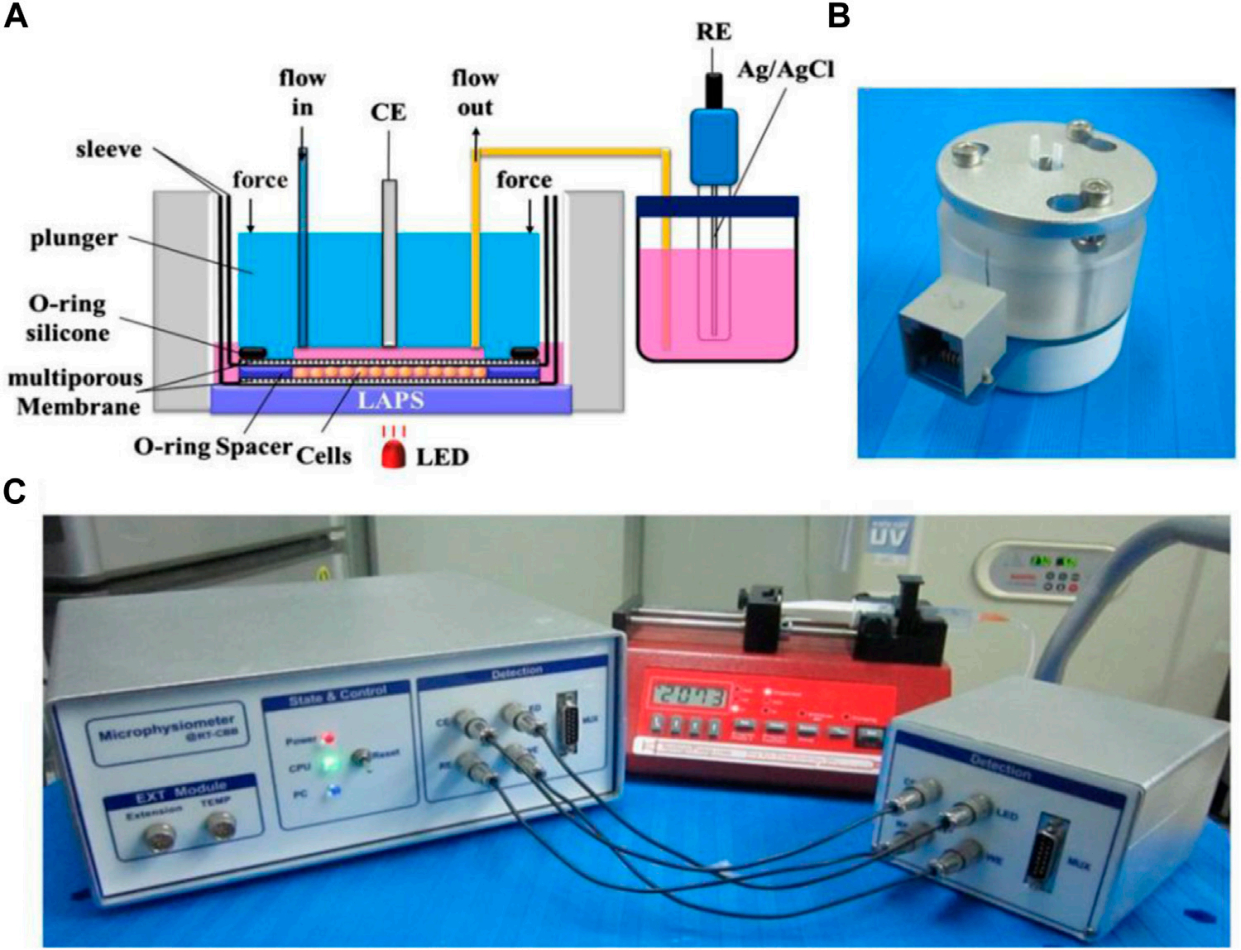
**(A)** Schematic structural diagram of cell microphysiology based on the LAPS **(B)** Microphysiometer fluid drive unit; **(C)** Schematic diagram of the overall structure of the detection system (Hu et al., 2013).

In addition, the LAPS chip has also been demonstrated to be suitable for monitoring cell activities stimulated by various drugs. For instance, Hu Ning et al. reported a LAPS analysis system for studying cellular metabolic mechanisms and drug reactions (Hu et al., 2013). As shown in Figure 5, by using gravity to drive cell culture solution and taking the LAPS as the substrate, the system constructs a microchannel cell culture chamber on its surface. The results show that the LAPS analysis system can detect cell metabolic activity in real time, and it is expected to become a practical platform for studying cell metabolic mechanism and drug evaluation.

**FIGURE 6:**
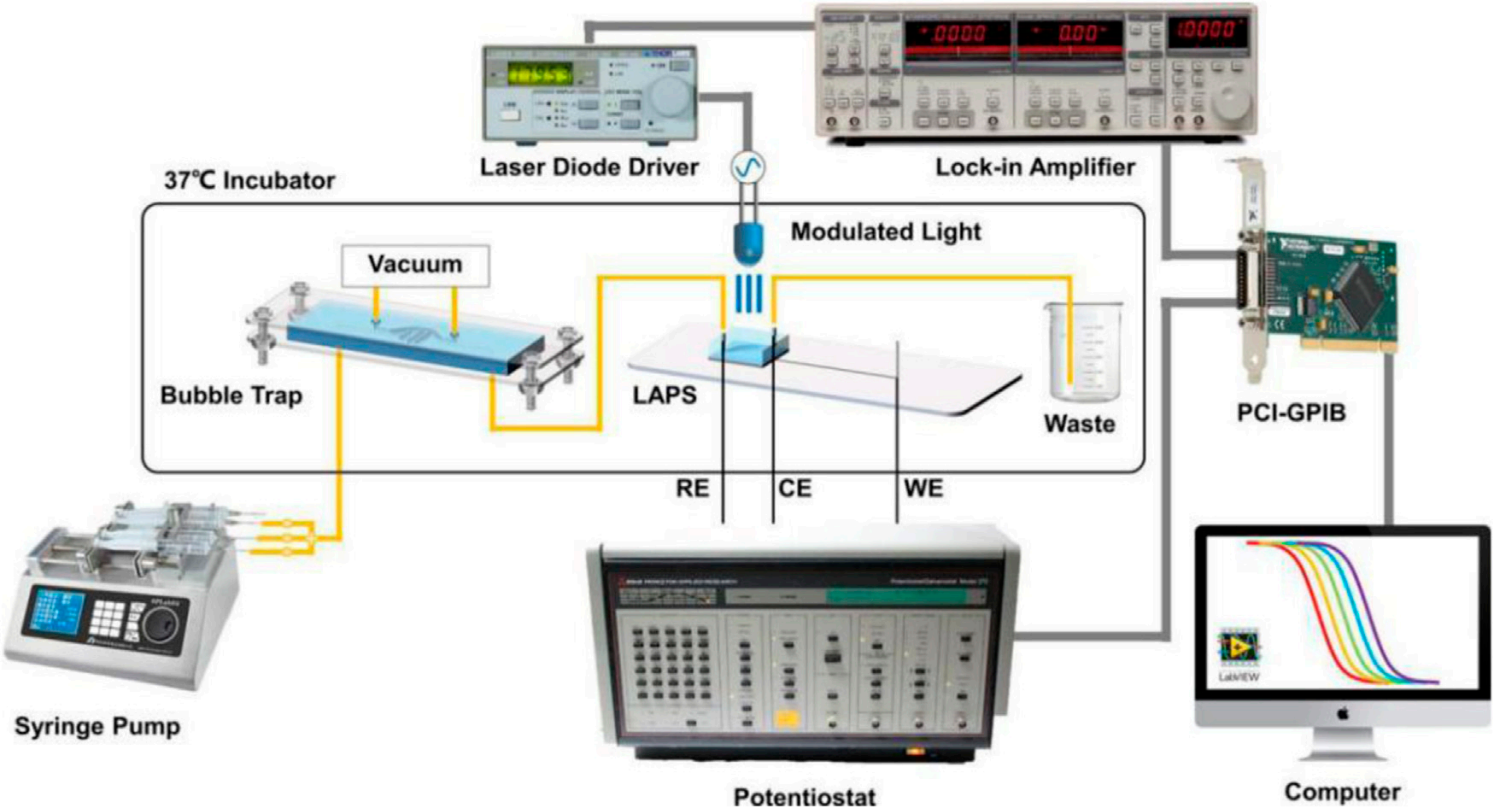
Schematic diagram of the microchannel detection system based on the LAPS(Liang et al., 2019b).

Microfluidic chips can provide a stable microenvironment for living cells, which greatly facilitates cell culture and measurement. Liang Tao et al. reported a LAPS analysis system for detecting the metabolic rate of liver tumor cells (Liang et al., 2019b). As shown in Figure 6, by using a syringe pump to drive microfluidics and setting a bubble capture structure in the microchannels, the interference of bubbles to the detection signal is effectively reduced. Because of its modular structure and high scalability, the LAPS analysis system has a good application prospect in organ chips.

**FIGURE 7:**
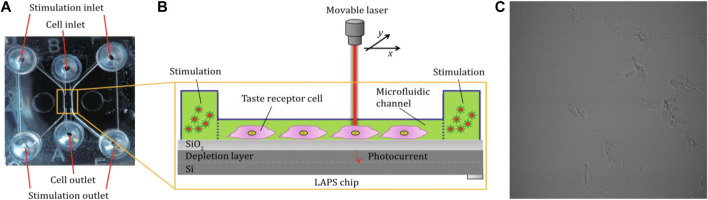
**(A)** Cell culture microanalysis system based on the LAPS. The cell culture chip includes two stimulation microcavities and one cell culture microcavity **(B)** The structure of the cell culture microcavity, which is combined with the LAPS to detect the change of membrane potential of taste receptor cells; **(C)** Cells in the microfluidic channel (Du et al., 2019).

The microfluidic chips are also integrated with LAPS chips to develop cell-based biosensors toward functional analysis of chemically sensitive cells. For example, Du Liping et al. reported a LAPS analysis system to detect the signal transduction mechanism of taste cells, which can realize the label-free functional analysis of bioengineering taste receptor cells *via* extracellular recording (Du et al., 2019). Extracellular potential changes of single bioengineered cells were recorded by the LAPS. Microfluidic chips were capable of providing stable microenvironments for cell measurements with a well-defined concentration stimulus. As shown in Figure 7, the LAPS can be used to detect the corresponding changes in cell membrane potential caused by specific bitter stimulation of taste cells. The LAPS analysis system is suitable not only for the determination of cells with specific functions but also for the study of chemical signal transduction mechanisms. However, this system was comparatively complex.

In addition to cells, the LAPS-integrated microfluidic chip is also used to measure the organ metabolism cultured on the surface of the LAPS chip. For example, Wu Qian et al. reported an organ-like chip system based on the LAPS (Wu et al., 2019). The circulation flow of culture solution in the organ-like chip was realized by means of microfluidics. Through the LAPS detection system, the changes of metabolites in the microenvironment of the organ culture solution were analyzed so as to realize the real-time monitoring of the physiological state of similar organs. Because the LAPS has a flat sensing surface, it is very suitable for cell microbial metabolism detection. In the future, more efforts should be made in miniaturization and reducing the cost of the detection system.

### Digital Droplet LAPS Microanalysis System

**FIGURE 8:**
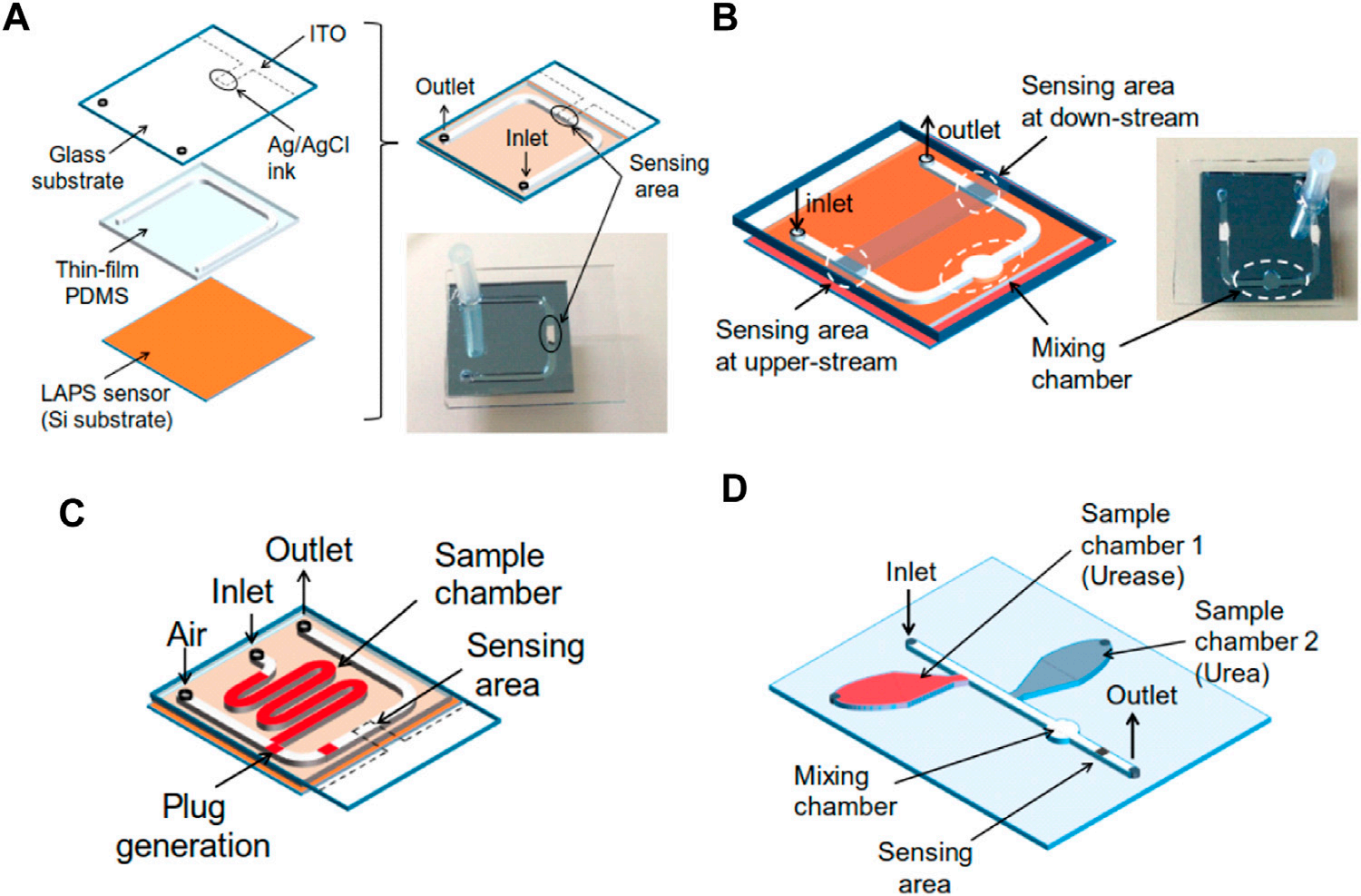
**(A)** Test structure of the microfluidic device combined with a LAPS; **(B)** Channel design with a chamber for merging and differential measurement; **(C)** Channel design to generate plugs on the chip; **(D)** Test structure with two sample chambers, one merging chamber and one sensing area (Miyamoto et al., 2016).

Digital droplet technology is an emerging one that greatly improves the capability of a microfluidic chip to deal with the small volume solutions. It has also been applied to LAPS-based microanalysis systems. For instance, Miyamoto et al. reported a microdroplet analysis system based on the LAPS (Miyamoto et al., 2016), as shown in Figure 8. The system uses a microinjector to add analytical droplets to the sample inlet of the microchannels, closes the electronic valve, opens the peristaltic pump, and the analytical solution is attracted into the microchannels to generate a droplet. When the droplet suction inlet is in full contact with the reference electrode, the peristaltic pump is closed (the position of the droplet is controlled by the opening or closing of the valve. Its moving speed is about 0.78 mm/s). The microdroplets are completed by opening the LAPS detection device, in which the minimum volume generated by the microelectrochemical analysis system is 400 nl. It is an interesting work to demonstrate the less solution consumed up to now, which could still decrease the volume of droplets and accuracy controlling them.

**FIGURE 9:**
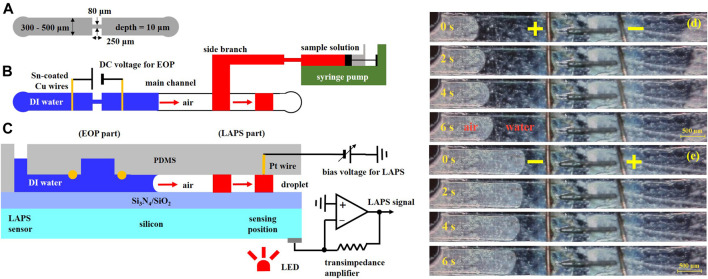
**(A)** Schematic diagram of the microchannel structure and size; **(B)** and **(C)** a top view and a side view of the microchannels. The microelectrochemical analysis system includes an electroosmotic driving section and a LAPS detection section. A microdroplet is generated near the “D" intersection of T-shaped microchannels. The microdroplet is pumped to the detection area of the LAPS by the electroosmotic micropump, and the microdroplet contacts with the Pt line to form a conduction loop to complete the detection of the microdroplet; EO flows in **(D)** right and **(E)** left directions (Li et al., 2016).

To address the problems that the volume is large, the structure is complex, the integration is difficult, and to complete the miniaturization process of the external pump, Li Xueliang et al. proposed the scheme to combine the LAPS which is used as a microfluidics detection sensor with an electroosmotic micropump to drive microfluidics (Li et al., 2016). The electroosmotic micropump does not have moving mechanical parts, and it is characterized by its simple structure, easy embedding into microchannels, no pulsation, and accurate transportation of microfluidics. Figure 9A shows a schematic diagram of the structure and size of the microchannels. Figures 9B,C show the top view and side view of the microchannels. T-shaped PDMS microchannels are constructed on the surface of the LAPS, and the branch channel uses a mechanical injection pump to transport the samples to be analyzed. Also, an electroosmotic micropump is embedded in the main microchannels, while the deionized water is used as the working fluid, and there is an air gap between the working fluid and the sample. The working principles of the microelectrochemical analysis system are as follows: first, through the injection pump, the sample analysis solution is transported to the “D” intersection of the T-shaped microchannels, and then, the injection pump is turned off. Second, the electroosmotic micropump is started to generate electroosmotic flow which would move to the right, and the sample analysis solution at the “D” intersection would be pumped to the downstream of the main microchannels to generate a microdroplet. Finally, when the microdroplets reach the detection area of the LAPS, the electroosmotic micropump is closed, and the LAPS detection system is started to complete the microdroplet detection.

**FIGURE 10:**
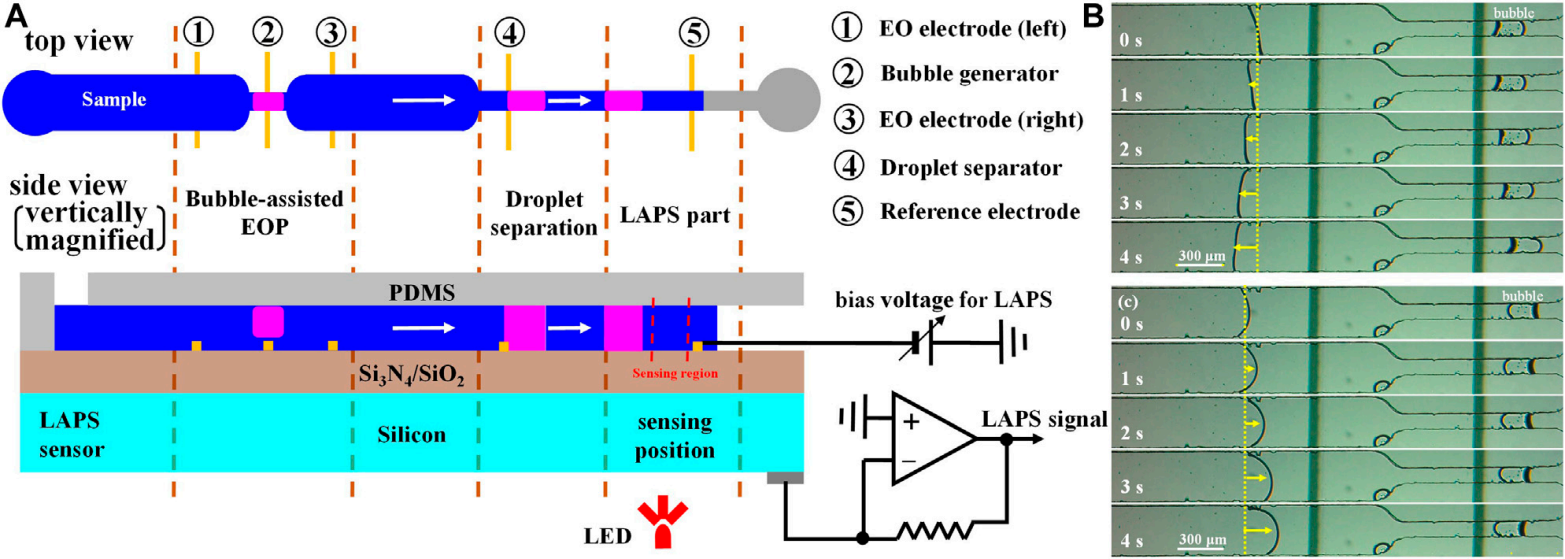
**(A)** Microelectrochemical analysis system with a LAPS as the sensing unit and a bubble-assisted electroosmotic micropump as the fluid driving unit; Bubble-assisted EO flows in **(B)** left and **(C)** right directions depending on the polarity of the pumping voltage (Li et al., 2017).

In order to improve the infusion efficiency of the electroosmotic micropump, Li Xueliang et al. proposed a technology of a bubble-assisted electroosmotic micropump and used it to construct a microelectrochemical analysis system (Li et al., 2017). As shown in Figure 10, the microelectrochemical analysis system includes three sections—a bubble-assisted electro-osmotic micropump section, a microdroplet separation section, and a LAPS detection section. Microchannels with a width of 80 μm and two Ti/Au electrodes (EO electrodes (right) and droplet separator) are added in the downstream of the bubble-assisted electroosmotic micropump, which perform functions as the droplet separator and reference electrode, respectively. The droplet separator has the same function as the bubble generator. A certain DC voltage is applied between the EO electrode (right) and droplet separator to generate bubbles through electrolysis so as to separate a certain volume of microdroplets. When the droplets are separated in the “bottleneck” area which is in the downstream of the microchannels, by a bubble-assisted electroosmotic micropump, the microdroplets are pumped to the detection area of the LAPS and contact with the reference electrode of the LAPS to complete the droplets’ detection. In summary, to deeply integrate the LAPS with microfluidics, the performance of microfluidic components and compatibility with the LAPS should be further improved.

## Conclusions and Prospects

Since the birth of the LAPS, it has been widely used in plenty of fields with its unique advantages. The research process of the LAPS involves many disciplines such as chemistry, biology, semiconductor, and electronic information, which is a typical interdisciplinary research field. In order to promote the wide application of the LAPS in biomedicine and other fields, in addition to improving the detection performance of sensors, the miniaturization of the detection system is also very important. A microchannel analysis system combining with the LAPS and microfluidic technology can not only reduce the consumption of biological and other valuable analysis samples but also realize the rapid measurement of multi-parameters in the dynamic process of complex chemical and biological reactions. This review summarizes the applications of the LAPS analysis system in ion diffusion, enzymatic reaction, microbial metabolic activity, and droplet microfluidics.

At present, the microchannel analysis system has been demonstrated to be suitable for applications requiring high surface flatness of sensors or a flexible definition of measurement areas, which show decisive advantages. However, the microchannel analysis system still has some limitations that hinder its further development and practical application. More efforts should be devoted to improve the performances of the microchannel analysis system to broaden their practical application fields. In the future, the development trend of the microchannel analysis system will focus on the following aspects: 1) how to improve the spatial resolution of LAPS records. The possible solutions may come from using novel semiconductor materials to manufacture LAPS chips, introducing more focused light spots, and laterally suppressing the movement of carriers in semiconductors. 2) How to speed up the imaging system. To solve this problem, it is necessary to utilize a high-speed scanning system, which allows rapid recording of data and rapid imaging from the sensor surface. 3) How to improve the measurement accuracy of the LAPS under microfluidic conditions. Since the measurements in the microscale or nanoscale solutions require very high accuracy, it is, thus, highly essential to improve the measurement accuracy of the LAPS, especially in the cases of high-throughput measurement. The possible solution to this problem may rely on the improvement of the peripheral circuits and data processing algorithms. In summary, it is necessary to further improve the spatial resolution, measurement speed, and accuracy of the LAPS to promote the wide applications of the microchannel analysis system in many fields such as biomedicine (Welden et al., 2021), food safety, and environmental protection (Tu et al., 2018; Tong et al., 2020; Li et al., 2021).
